# Does ambient noise or hypobaric atmosphere influence olfactory and gustatory function?

**DOI:** 10.1371/journal.pone.0190837

**Published:** 2018-01-25

**Authors:** Torsten Rahne, Robert Köppke, Michael Nehring, Stefan K. Plontke, Hans-Georg Fischer

**Affiliations:** 1 University Hospital Halle (Saale), Department of Otorhinolaryngology, Head and Neck Surgery, Martin Luther University Halle-Wittenberg, Halle (Saale), Germany; 2 Air Force Centre of Aerospace Medicine, Aviation Physiology Training Centre, Königsbrück, Germany; 3 Military Hospital Hamburg, Department of Otorhinolaryngology (ENT), Head and Neck Surgery, Hamburg, Germany; University of Graz, AUSTRIA

## Abstract

Multidimensional food perception is based mainly on gustatory and olfactory function. Recent research has demonstrated that hypobaric pressure impairs gustatory function and that background noise or distracting auditory stimulation impairs olfactory function. Using a hypobaric chamber, the odor identification, discrimination, and thresholds as well as taste identification and threshold scores were measured in 16 healthy male volunteers under normal and hypobaric (6380 ft) conditions using clinically validated tests. In both conditions, background noise was either canceled out or replaced by white noise presentation (70 dB sound pressure level). Olfactory sensitivity for n-butanol and gustatory sensitivity were impaired in a hypobaric atmosphere. White noise did not influence the odor test results. White noise stimulation impaired sensitivity for sour and sweet but not for bitter or salty tastants. We conclude that hypobaric or noisy environments could impair gustatory and olfactory sensitivity selectively for particular tastants and odorants.

## Introduction

Why do food tastes differ or decrease in airborne craft? This question is of interest especially for flight staff, passengers, and pilots. The due to to low air pressure hypobaric atmosphere in a cabin has been hypothesized to be the main contributing factor in a decreased sense of flavor [1,2], but this sense is a multisensory perception built on gustatory (tasting), olfactory (smelling), and sometimes oral-somatosensory (feeling) inputs [3]. Maier et al., e.g., demonstrated functional projections from primary taste to the primary olfactory cortex in rats [4]. Spence reviewed the growing evidence on multisensory flavor perception and identified visual, auditory, and trigeminal contributions to this sense [5]. The ability to smell and taste contributes most to the perception of flavors, so both olfactory and gustatory function should be assessed in flavor perception studies, including those evaluating high-altitude effects.

Years ago, Bert reported decreased olfactory function at high altitudes under low air pressure [6]. Integral chemosensory perception is reported to be affected by a hypobaric environment, leading to decreased taste function [7,8]. Kühn et al. systematically investigated the effect of air pressure on butanol odor threshold and found an increased sensitivity under hyperbaric conditions and a decreased sensitivity under hypobaric conditions compared to atmospheric pressure [2]. Burdack-Freitag et al. reported higher taste and odor thresholds at low atmospheric pressure and noise compared to subsequently applied normal pressure [1]. A reduced sense of olfaction under these conditions not only contributes to a reduced perception of flavor but also could be dangerous. If smoke or electrical odors go undetected, the risks for personnel and passengers could be increased.

In a flying aircraft, cabin noise is present in addition to reduced air pressure, and auditory stimulation also influences flavor perception [9]. Thus, both effects could contribute to decreased tasting or smelling in this special environment. According to the so-called sensory dominance hypothesis [10], auditory information dominates oral-somatosensation. The type, tempo, and loudness of background music influence the human perception of food, as well [9]. However, most studies have focused on the psychological attributes of auditory cues. Woods et al. have shown, for example, that noisy environments reduce crunchiness but not flavor strengths [11]. Seo et al. found no effect of background noise on odor perception tasks [12], whereas Velasco et al. did [13]. Yan and Dando simulated real aircraft cabin noise and found a reduced intensity rating for sweet tastants but not for salty, sour, and bitter [14]. Studies assessing the influence of noise on flavor perception have largely focused on psychometric endpoints and not on clinically applied outcome measures [15].

We assessed the effects of noise and reduced air pressure on olfactory and gustatory function in a prospective, randomized study in a well-defined experimental setting. To clearly separate the two effects, clinically standardized test materials were applied, i.e., the ‘Sniffin Sticks’ test battery [16–18] and ‘Taste Strips’ [19].

## Materials and methods

Sixteen men ages 19 to 32 years (mean, 23.4 years; SD, 3.5 years) in good health with no known hearing, olfactory, or gustatory disorders were recruited as volunteers. All participants provided written informed consent, had study-related insurance, and were paid for their participation. The study was approved by the Ethics Committee of the Martin Luther University Halle-Wittenberg and in accordance with the Declaration of Helsinki.

Prior to study measurements, a medical examination was performed to evaluate for cardiovascular (heart) and pulmonary (lung) diseases. Additionally, an ear–nose–throat examination was performed. Middle ear pressure equalization was checked by microscopic ear inspection with the Valsalva maneuver and tympanometry. To exclude pathological anatomy of the inner nose or signs of rhinosinusitis, i.g, due to a cold, a nose endoscopy was performed. To ensure good hearing, pure-tone audiometry was performed in all participants. Exclusion criteria were active smoking, claustrophobia, odor or tastant allergies, and current use of streptomycin, D-penicillamine, diltiazem, nifedipine, amitriptyline, methotrexate, amphetamines, alcohol, local vasoconstrictive substances, strychnine, codeine, or lidocaine.

Study measurements were performed in the custom-made ‘High Altitude and Climate Chamber’ of the German Air Force at Königsbrück, Germany, with a volume of 59 m^3^. Blood oxygen concentration (pO_2_) and electrocardiogram output were monitored and recorded during the measurement sessions.

For hypobaric measurements, the pressure was set to 800 mbar (6380 ft), i.e., similar to the cabin pressure of commercial aircrafts (HYPO conditions). As the control condition, a normal pressure of 950 mbar (1760 ft) was used (NORMAL conditions). The participants were either continuously exposed to MATLAB-generated white noise with a sound pressure level of 70 dB by E-A-RTONE 3A-insert earphones (3M, St. Paul, MN, USA) (NOISE conditions), or protected from the cabin noise by E-A-R earplugs (3M, St. Paul, MN, USA) and SPERIAN circumaural (Howard Leight, Smithfield, RI, USA) hearing protectors (SILENCE conditions). This sound pressure level is equal to the loudness of city traffic. The intrinsic noise level of the chamber was 59 dB A-weighted sound pressure level and thus less than the loudness of conversational speech. The temperature (20.5°C ± 0.5°C), humidity (44% ± 2%), oxygen concentration (20.8% ± 0.2%), and carbon dioxide concentration (0.055% ± 0.015%) were kept constant. The odor state of the cabin was kept stable, and complete air exchange was performed after every measurement.

For every combination of noise and pressure condition in a randomized order, olfactory and gustatory function was measured in a randomized order, including the subsets. The eyes were masked. Olfactory function was evaluated using the identification, discrimination, and threshold subtests of the ‘Sniffin Sticks’ (Burghart, Wedel, Germany) test battery [16]. Therefore, odorants were presented in commercially available felt-tip pens with a lengths of 14 cm with an inner diameter of 1.3 cm. Instead of liquid dye the tampon was filled with liquid odorants or odorants dissolved in propylene glycol, to the total volume of 4 ml. After cap removal, odor-dispensing felt-tipped pens were placed in front of each participant’s nostrils for about 3 seconds. The participants wore eye patches, and the investigators wore cotton gloves.

For measuring odor discrimination, 16 triplets of pens were presented in a randomized order. The participants had to decide which of the pens of each triplet contained a different odor compared to the other two pens. The sum of correct discriminations was the discrimination score, ranging from 0 to 16.

The identification task for odors consisted of a forced-choice identification of 16 successively presented odors. For every pen, four choices were possible. The sum of correct responses was the identification score, ranging from 0 to 16.

Olfactory thresholds were measured by presenting pens containing a varying dilution of n-butanol [16]. One of each presented triplet of pens contained the solution, which had to be identified by the participants as the forced-choice task. Starting from a high concentration, the concentration was reduced until a false response was reported by the participants. Then, the concentration was incrementally increased until a correct response was reported. After six reversals, the mean of the last four reversals was defined as the olfactory threshold score, ranging from 1 (highest concentration) to 16 (lowest concentration). A cumulative score (TDI, [16]) was calculated for the threshold, identification, and discrimination tasks for every combination of noise and pressure conditions separately.

Gustatory function was measured based on filter paper strips [19,20] (Burghart, Wedel, Germany), with a tip area being impregnated with the tastant (four concentrations each of the four basic taste qualities of sweet (sucrose), salty (sodium chloride), sour (citric acid), and bitter (quinine hydrochloride). Two control strips without tastants were included in the test, too. The strips were placed in random order on the tongue. The participants had to choose one of the four flavors or ‘no flavor.’ The sum of correct responses was the gustatory score, ranging from 0 to 18.

Statistical analyses were carried out using SPSS 23 software (IBM, Ehningen, Germany). If normally distributed (nonsignificant Shapiro-Wilk-Test) cumulative TDI score, identification score, discrimination score, and olfactory threshold were compared using repeated measures ANOVAs with the within-subject factors pressure (HYPO, NORMAL) and noise (NOISE, SILENCE). Mean gustatory scores were compared using repeated measures ANOVA with the within-subject factors quality (SWEET, SOUR, SALTY, BITTER), pressure (HYPO, NORMAL) and noise (NOISE, SILENCE). Degrees of freedom were reduced using Greenhouse-Geisser correction if the Mauchly test was significant. For post hoc analysis, least significant difference tests were applied. For all comparisons, α was set to 95%.

## Results and discussion

In one participant (ID4), the olfactory threshold could not be measured because even the highest odor concentration could not be identified. Participants were able to complete all other measurements. Table 1 shows the mean TDI scores as well as the gustatory threshold score for all conditions. In all participants, the pure-tone hearing loss for air conduction was ≤15 dB HL for frequencies from 125 Hz to 8000 Hz, indicating normal hearing. Nose function was normal, i.e., no acute or chronic sinusitis or polyposis was present. Mean pO_2_ was 96.3% (SD: 1.8%) in the NORMAL conditions and 94.7% (SD: 2.2%) in the HYPER conditions.

**Table 1:** Mean scores of olfactory and gustatory function for all conditions.

	NORMO		HYPO	
	SILENCE	NOISE	SILENCE	NOISE
*Objective*	Mean (SD)	Mean (SD)	Mean (SD)	Mean (SD)
***Olfactory function***				
**Identification score**	13.31 (1.38)	13.44 (1.03)	13.31 (1.53)	14.06 (0.77)
**Discrimination score**	12.5 (1.78)	12.06 (2.62)	12.81 (1.87)	12.31 (1.49)
**Threshold score**	8.81 (1.72)	9.03 (1.46)	8.39 (1.48)	8.00 (1.51)
**TDI**	34.34 (2.81)	34.23 (3.16)	34.25 (3.21)	34.27 (2.56)
***Gustatory function***				
**Gustatory score**	11.06 (2.74)	11.19 (2.64)	10.25 (2.98)	10.38 (2.59)
**Sweet**	2.88 (0.72)	3.00 (0.89)	2.56 (1.03)	3.13 (0.86)
**Sour**	2.44 (0.89)	2.06 (0.85)	2.62 (0.72)	2.13 (0.81)
**Salty**	2.62 (1.15)	2.88 (0.80)	2.31 (1.20)	2.00 (1.16)
**Bitter**	3.13 (1.31)	3.25 (1.07)	2.75 (1.34)	3.13 (0.81)

**Fig 1:**
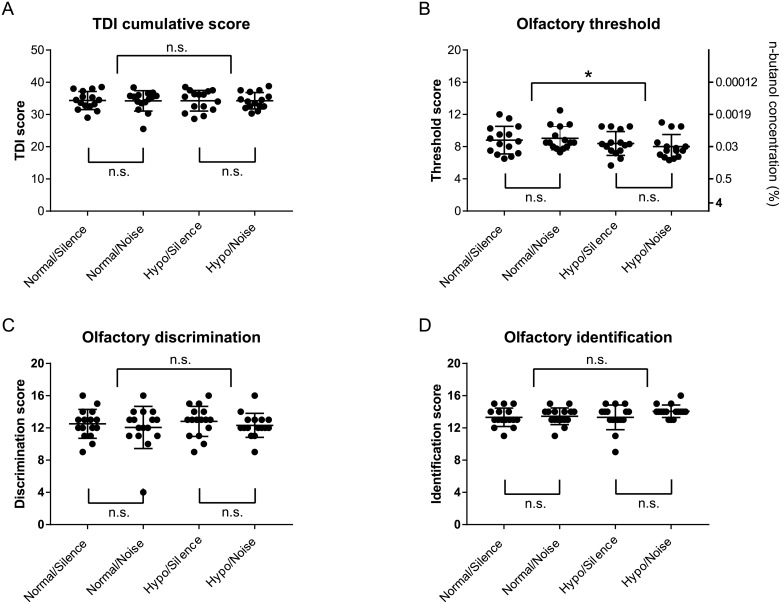
Individual and mean (±SD) olfactory function scores based on ‘Sniffin Sticks’. (A) Cumulative TDI, (B) threshold, (C) discrimination, and (D) identification scores. * Significant differences at p < 0.05 (ANOVA), n.s.: not significant.

Fig 1 shows the cumulative olfactory TDI score and the underlying subscores for odor identification, discrimination, and olfactory threshold. Noise or pressure had no significant effect on the mean TDI score and showed no interaction. Pressure significantly influenced mean olfactory threshold (F(1,14) = 12.9, p < 0.01), but noise did not, and the two factors showed no interaction. Pressure and noise also had no significant effect on mean identification and discrimination scores. Post hoc comparisons showed a significantly increased mean threshold in the HYPO (8.20 ± 0.35) compared to the NORMAL condition (8.92 ± 0.37).

**Fig 2:**
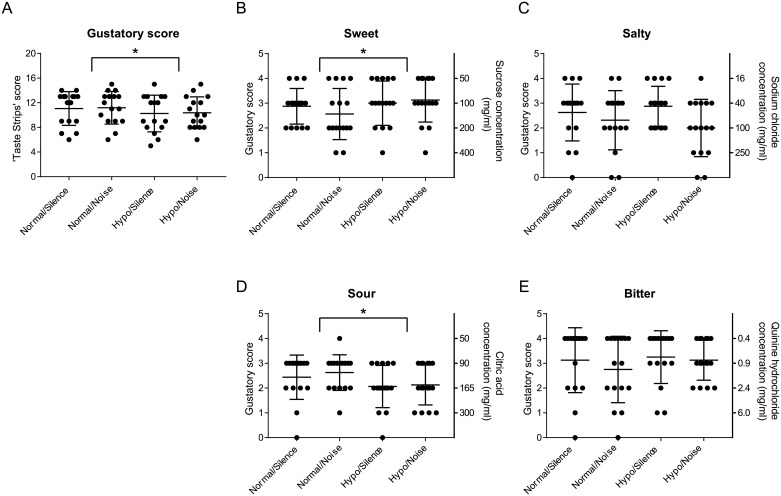
Gustatory score and underlying tastant thresholds for all participants. (A) Individual and mean (±SD) cumulative function scores based on ‘Taste Strips’ identification. (B–E) The underlying thresholds for the different tastants as well as the corresponding tastant concentration are also shown. * Significant differences at p < 0.05 (ANOVA).

Fig 2 shows the gustatory score and underlying tastant scores for all participants. Pressure (F(1,15) = 5.8, p < 0.05) and quality (F(3,45) = 7.7., p < 0.01) significantly affected the mean gustatory score, but noise did not (F(1,15) = 0.1, p > 0.05). Only the factors noise and quality showed a significant interaction (F(3,45) = 3.2, p < 0.05. Significantly decreased scores were found for SOUR compared to SWEET and BITTER and SALTY compared to SWEET and BITTER. Under the NOISE condition, mean scores for SWEET were significantly increased, but mean scores for SOUR were significantly reduced. No other effects and interactions were observed.

For all conditions, the results show TDI scores within normative ranges for age and sex [17]. In addition, the subscores for identification, threshold, and discrimination were within normative ranges; however, the effect of hypobaric atmosphere on the olfactory thresholds was significant. One possible reason for decreased olfactory sensitivity may be reduced odor concentration following gas expansion under low pressure. Because the odor concentration in the identification and discrimination tasks was clearly above threshold, the hypobaric atmosphere did not affect the respective subscores. We tested the threshold using only one odor (n-butanol), which obviously is not representative of daily life smell perception. As Burdack-Freitag et al. reported [1], sensitivity for many other odors will decrease in a hypobaric atmosphere, and safety-related alert systems in hypobaric environments should be based on automatic sensors.

The hypobaric atmosphere significantly decreased cumulative gustatory function. The test material contains strips with different tastant concentrations, so the score actually reflects a measurement of the gustatory thresholds. To emphasize the threshold aspect of gustatory function testing, we included the response ‘no taste’ among the possible choices; therefore, our scores are somewhat smaller that the normative values reported previously [19].

Hypobaric atmospheres may cause hypoxia, i.e., a reduced pO_2_, in the blood, which could also affect olfactory and gustatory functions [21]. In this study, however, pO_2_ was decreased only slightly in the hypobaric condition compared to normal pressure. Perhaps at greater pressure differences, the effect of hypoxia on olfactory and gustatory perception might be more prominent. The observed little hypoxia was not expected to interact with the effects of ambient noise on olfactory and gustatory outcomes in this study.

The effect of the hypobaric atmosphere on olfactory and gustatory functions was rather small but significant. The sensitivity increment for n-butanol (threshold) was in the same order as that observed by Kühn at al. [2]. Also in line with earlier findings [2], we observed no effect of pressure on odor discrimination, but the gustatory score was significantly reduced. As with previous results [1], the higher taste thresholds for salty drove the gustatory score reduction. The lower (better) threshold for sweet, however, is discordant with earlier findings [1], whereas the threshold constancy of sour and bitter tastants is in line with those results. It may be that the different flavor application methods (diluted in drinking water vs. Taste Strips) influenced the results. Also physical effects caused by the different air pressure atmospheres could have manipulated the Sniffin’ sticks delivery system. In addition, in the current study the pressure difference between the hypobaric and normal atmospheres was rather small. Other psychologically relevant effects may be more important to reduced taste and smell perception in aircraft.

We found no general influence of noise on olfactory and gustatory functions but did identify an effect for sweet and sour, with opposing trends. Related findings are mixed in the literature: Seo et al., for example, found no effect of noise types on odor perception [12], but others have [11]. As a relationship between ratings of the likings of background noise and rating of the food was found [11], we assume that sound-induced emotion or a perceptional mismatch between sound and taste might underlie changes in food tastes. The white noise used in our study is a rather flat sound with no emotional valence and should therefore have had no effect on olfactory and gustatory function.

In our opinion, the reported cross-modal effects between auditory and taste perceptions [15,22] operate at a cortical level and strongly depend on the psychoacoustic features of the sound in relation to the food. In this study, artificial tastants (no food) and sound were used and therefore might not have evoked a cross-modal effect. The hypothesis that this effect arises from mechanostimulation of the chorda tympani nerve, which transits directly across the tympanic membrane of the middle ear [14], cannot be addressed with this study but should be in further clinical studies.

## Conclusions

In conclusion, we found a small but significant reduced gustatory and olfactory sensitivity in a hypobaric atmosphere compared to normal pressure but no general effect of ambient noise. White noise decreased sensitivity for sour and increased it for sweet tastants. This effect would be expected to be greater in increased pressure differences and thus be relevant for pilots, high-altitude climbers, or workers in hypobaric conditions. For travelers in aircrafts, other factors such as humidity, temperature, stress, or lighting might be more relevant than ambient noise for an altered perception of flavors.

## Supporting information

S1 File Individual results for taste and smell tests. (TXT)
